# Ellagic Acid Alleviates Abnormal Fat Reduction by Activating the RXRβ–PPARγ Pathways in a CT26 Tumour‐Induced Cachexia Mouse Model

**DOI:** 10.1002/jcsm.70176

**Published:** 2026-01-28

**Authors:** Woo Yong Park, Beomsu Kim, Gahee Song, Sang Hee Kim, Jin‐Hyung Kim, Ja Yeon Park, Se Jin Jung, Wenjun Jiao, Jisoo Han, Taekyoung Kong, Kwang Seok Ahn, Hyun Jeong Kwak, Jae‐Young Um

**Affiliations:** ^1^ Department of Pharmacology, College of Korean Medicine Kyung Hee University Seoul Republic of Korea; ^2^ Kyung Hee Institute of Convergence Korean Medicine Kyung Hee University Seoul Republic of Korea; ^3^ Department of Science in Korean Medicine, Graduate School Kyung Hee University Seoul Republic of Korea; ^4^ Department of Biomedical and Pharmaceutical Science, College of Pharmacy Kyung Hee University Seoul Republic of Korea; ^5^ Department of Bio and Fermentation Convergence Technology Kookmin University Seoul Republic of Korea

**Keywords:** abnormal fat reduction, cancer cachexia, ellagic acid, PPARγ, RXRβ

## Abstract

**Background:**

Cancer cachexia is a syndrome characterized by significant weight loss, particularly of adipose tissue, which negatively impacts patient survival. Pharmacological approaches to prevent this abnormal fat reduction remain poorly defined. This study investigated the potential of ellagic acid (EA) to mitigate cancer‐induced fat loss.

**Methods:**

In vitro, 3T3‐L1 adipocytes were exposed to 50% conditioned medium (CM) from CT26 colon cancer cells to mimic cachectic conditions. The effects of EA on lipid accumulation and adipogenesis‐related markers (PPARγ and RXRβ) were analysed. The mechanism was validated using molecular docking and siRNA‐mediated gene silencing. In vivo, a cachexia mouse model was established by subcutaneous injection of CT26 cells into BALB/c mice. Mice were orally administered EA at dose of 10, 20 and 40 mg/kg, respectively (*n* = 7 per group) for 14 days to evaluate its protective effects.

**Results:**

In 3T3‐L1 cells, the 50% CM‐treatment reduced lipid accumulation by 14% (*p* < 0.05) and downregulated PPARγ (*p* < 0.05) and RXRβ (*p* < 0.01) expression. The EA treatment restored diminished lipid levels and rescued downregulated the expression of PPARγ (*p* < 0.05) and RXRβ (*p* < 0.01). This pro‐adipogenic effect was abolished by siRNA‐mediated inhibition of RXRβ. In the cachexia mouse model, EA treatment significantly improved physical performance and body composition. Specifically, EA enhanced grip strength by 1.32‐fold (10 mg/kg, *p* < 0.0001) and 1.24‐fold (20 mg/kg, *p* < 0.001), increased the inguinal white adipose tissue (iWAT) mass by 59.8% (10 mg/kg, *p* < 0.01), and 56.1% (20 mg/kg, *p* < 0.05), and was accompanied by a significant increase in the tumour‐free body weight of 6.4% (10 mg/kg, *p* < 0.05), and 5.6% (20 mg/kg, *p* < 0.01) compared to cachectic mice. EA treatment did not affect tumour growth. It increased the adipogenesis‐related protein expression of C/EBPα (2.1‐fold, *p* < 0.05), PPARγ (1.5‐fold, *p* < 0.05) and SREBP1 (1.5‐fold, *p* < 0.05) in the iWAT. The EA treatment enhanced the nuclear localization index of RXRβ in the iWAT by 0.51 (10 mg/kg, 1.9‐fold, *p* < 0.05) and 0.46 (20 mg/kg, 1.7‐fold, *p* < 0.05) compared to cachectic mice.

**Conclusions:**

EA mitigates cancer cachexia‐induced fat loss by the activation of the RXRβ–PPARγ pathway. These findings present EA as a potential pharmacological agent to improve the abnormal fat reduction and muscle dysfunction associated with cachexia.

## 1 Introduction

Cachexia is an irreversible multi‐organ syndrome identified by significant weight loss, primarily affecting skeletal muscle, adipose tissue and organs, including the bones, brain, liver, gut and heart [1]. This state occurs due to an interplay of metabolic dysfunctions, inflammatory responses and changes in an organism's ability to utilize nutrients [1]. Cancer cachexia occurs in approximately 50%–80% of cancer patients and contributes to up to 20% of cancer‐related deaths [2]. Cancer cachexia is particularly prominent in patients with gastrointestinal and pancreatic cancers, where about one‐third of the patients lose at least 10% of their baseline weight [3]. However, practical and ethical challenges make human studies on cancer cachexia difficult to conduct. As a result, rodent models using xenograft tumours are widely used to investigate the mechanisms underlying muscle and fat wasting [4]. Additionally, pharmacological interventions targeting cancer cachexia present challenges in evaluating their effect due to uncertainty in determining whether candidate compounds alleviate cachexia indirectly by inhibiting tumour growth or directly by preventing muscle and fat wasting.

Adipose tissues play a crucial dual role in energy metabolism, acting as reservoirs for lipid storage and endocrine organs that release hormones and adipokines [5]. The interaction of these molecules with various organs has a crucial role in regulating numerous physiological processes [6]. In the context of cancer cachexia, observations have indicated that increased lipid utilization from adipose depots in tumour‐bearing cachectic mice points to a notable depletion of lipid reserves in adipose tissues, leading to decreased fat mass [7]. Peroxisome proliferator‐activated receptor gamma (PPARγ) is a crucial nuclear receptor that modulates the pathways involved in de novo fatty acid (FA) and triglyceride synthesis [8]. In the context of cachectic adipose tissue, a decrease in both mRNA and protein levels for PPARγ has been noted [9]. However, the precise mechanism by which it suppresses adipose PPARγ expression is not fully understood. Clarifying this is important, as endogenous PPARγ ligands are necessary to address metabolic disturbances in adipose tissues. Key regulators such as retinoid X receptors (RXRs), liver X receptors (LXRs) and PPARγ coactivator 1 α (PGC1α) play a significant role in the regulation of PPARγ expression within the adipose tissue affected by a tumour [9, 10]; nevertheless, their specific functions are not fully elucidated. Thus, regulating PPARγ and PGC1α has been suggested as a potential strategy to rescue the dysfunction of adipose tissue associated with cancer cachectic malnutrition; however, the mechanisms remain unclear.

Ellagic acid (EA), a naturally occurring polyphenolic molecule, is present in berries [11]. EA has received considerable research attention due to its wide array of biological properties, including its antioxidant, anti‐inflammatory and anti‐cancer activities [12]. It has been reported that EA could control mitochondrial biogenesis and OXPHOS activity in various experimental models, including acute cold‐exposed mice, high fat diet (HFD)‐mediated obese mice and androgen‐stimulated benign prostate hyperplasia (BPH) rats [11, 13, 14]. Briefly, EA treatment in cold exposure and HFD conditions could increase mitochondrial biogenesis, mitochondrial dynamics and mitochondrial uncoupling, contributing to restoring metabolic health. However, EA disrupts the enterocyte cell cycle and mitochondrial ATP production, thereby preventing BPH. Based on these observations, we hypothesized that EA treatment restores altered metabolism in adipose tissues by reducing anabolic pathways, including lipogenesis, and elevating catabolic pathways such as lipolysis and mitochondrial uncoupling in cancer cachexia. To investigate the effect of EA on adipose tissue depletion in tumour‐bearing mice, we selected a lower EA dosage (10 mg/kg), which was administered by injection into the lateral flank of mice to prevent interference with tumour expansion. Indeed, numerous studies have shown that cancer treatment for EA uses high dosages ranging from 40 to 100 mg/kg [15, 16]. Therefore, the present study examined the protective effect of EA on fat loss, focusing on how to regulate lipid metabolism by EA treatment, using the colon tumour 26 (CT26)‐induced cancer cachexia mouse model and white‐differentiated 3T3‐L1 cells.

## 2 Materials and Methods

### 2.1 Animals and Experimental Design

Male 6‐week‐old BALB/c mice were purchased from Daehan Biolink Co. (Eumsung, Korea) and maintained for 1 week prior to the experiments with a Rodent NIH‐41 Open Formula Diet with 5% fat (Central Lab. Animal Inc, Seoul, South Korea) and water ad libitum. Cancer cachexia was induced as described in our previous study [17]. Briefly, all mice were first randomized by body weight and divided into a non‐tumour‐bearing vehicle group, which received a subcutaneous injection of PBS, and a tumour‐induction group, which was injected with 5 × 10^5^ CT26 colon cancer cells (CRL‐2638, ATCC, Rockville, MD, USA). One week following the tumour cell injection, the tumour‐bearing (CT26) mice were re‐randomized based on their tumour volume and body weight. They were allocated into four distinct subgroups: a vehicle control group receiving 0.9% normal saline and three experimental groups receiving EA at doses of 10, 20 or 40 mg/kg. All treatments were administered orally 5 days a week for a total of 3 weeks. The body weight of mice was measured weekly.

Tumour size was calculated every week based on the formula width × length using an Absolute digimatic caliper (Mitutoyo, Kawasaki, Japan). The relationship between tumour weight and volume can be calculated using the formula 0.52 × tumour length × tumour width^2^ according to our previous study [17]. After 3 weeks, the mice were euthanized by cervical dislocation under CO_2_ asphyxiation, 24 h after the final drug administration. The tissues were collected, weighed and kept frozen at −80°C. All procedures in the animal experiments were performed after receiving approval from the Animal Care and Use Committee of the Institutional Review Board of Kyung Hee University [KHUASP (SE)‐21‐311]. In vivo experiments were conducted in a blinded manner with respect to treatment allocation for each mouse. At sacrifice, bilateral tibialis anterior (TA), gastrocnemius (GAS) muscles, and inguinal white adipose tissue (iWAT) and epididymal white adipose tissue (eWAT) were dissected and weighed.

### 2.2 Grip Strength Test

An electronic dynamometer was used to measure grip strength (#BIO‐GS4, BIOSEB, FL, USA). Mice were placed on the dynamometer with the forelimbs grabbing the metal strip and gently pulling back the tail in the horizontal direction, then increasing the force gradually until the mice were removed. Three measured values were recorded and averaged.

### 2.3 Adipokine Array

3T3‐L1 cells were treated with conditioned medium (CM), followed by treatment with EA. After the incubation period (Day 6), the culture supernatant was collected and centrifuged at 500 ×*g* for 5 min at 4°C to pellet dead cells and other fragments. The cell‐free supernatant was then collected for further analysis. A mouse adipokine array was performed using a commercial Proteome Profiler Mouse cytokine array Kit (#ARY006, R&D Systems, Minneapolis, MN, USA) according to the manufacturer's instructions.

### 2.4 Protein Extraction and Western Blot Analysis

The cells and tissues were lysed in cell lysis buffer (#9803, Cell Signaling Technology, Danvers, MA, USA) on ice for 30 min. Insoluble debris was discarded by centrifugation at 13 000 rpm for 30 min. The protein concentration was measured with the Pierce BCA Protein Assay Kit (#23225, Thermo Fisher Scientific). The lysates (20 μg) were separated by sodium dodecyl sulphate‐polyacrylamide gel electrophoresis (SDS‐PAGE) and transferred onto a polyvinylidene fluoride membrane (Merck, Darmstadt, Germany). The membranes were blocked in 3% BSA and then incubated with the respective primary antibody (1:1000) overnight at 4°C, followed by incubation with horseradish peroxidase (HRP)‐conjugated secondary antibody (1:10000) for 1 h at room temperature. The protein signals were detected using the ECL advance kit. The chemiluminescent intensities of the protein signals were quantified using the Image J software program (National Institute of Health, MD, USA). The following primary antibodies were used: PPARγ (Cat# 2435S, Cell Signaling Technology), RXRβ (sc‐741, Santa Cruz Biotechnology) and SREBP1 (sc‐366, Santa Cruz Biotechnology). Detailed information regarding these antibodies is provided in Table S2.

### 2.5 BODIPY Staining

To visualize and quantify intracellular lipid accumulation, cells were stained with BODIPY 493/503 (#D3922, Thermo Fisher Scientific). The cells were stained with 2 μM of BODIPY for 30 min at 37°C, fluorescence was visualized using the EVOSR Cell Imaging System (Thermo Fisher Scientific), and lipid droplets were quantified with ImageJ software [S3].

### 2.6 Flow Cytometry

The cells were fixed with 10% formalin for 1 h. Fixed cells were permeabilized with 0.05% Triton X‐100 solution for 10 min, and then were blocked with PBS containing 3% BSA for 30 min. For staining SREBP‐1, cells were incubated with SREBP‐1 (1:50) for 1 h, followed by incubation of a secondary antibody conjugated to Alexa Fluor 647 for 30 min. DAPI (1:500) was used nuclear staining. After washing with PBS, the cells (1 × 10^5^/mL) were analysed with the Attune NxT Flow cytometer (Thermo Fisher Scientific), and were recorded 5000 events per group [S3].

### 2.7 Haematoxylin and Eosin Staining

The tissues were fixed in 10% formalin and embedded in paraffin. Embedded tissues were cut into 4 μm sections, and the tissue sections were deparaffinized with xylene and rehydrated in ethanol/water. The sections were stained with H&E and then examined with the EVOS M7000 Imaging System (Thermo Fisher Scientific). Lipid size was calculated using the ImageJ software program (National Institute of Health, Bethesda, MD, USA) [11].

### 2.8 Immunofluorescence Staining Assay

The tissue sections were deparaffinized with xylene and rehydrated in ethanol/water. The tissues were blocked with 5% BSA solution and incubated with the indicated primary antibodies (anti‐SREBP‐1, 1:50 in 5% BSA) overnight at 4°C. After washing, the tissue sections of WAT were incubated with Alexa Fluor 488‐conjugated secondary antibody (1:1000), and fluorescence images were taken using a Fluoview FV1000 confocal microscope (Olympus, Tokyo, Japan). The green fluorescence intensity (Alexa Fluor 488) in each image was quantified with the ImageJ software and analysed by GraphPad Prism Version 8 (GraphPad Software, San Diego, CA, USA) [11].

### 2.9 Protein–Molecule Binding Affinity Prediction

The protein 3D crystal structure of RXR beta was obtained from the RCSB PDB database (PDB ID: 7A78, resolution: 1.72 Å). To perform molecular docking, the ligands and water were removed, and then hydrogen atoms were added. The 3D structure of the ligand, EA, was minimized using the force field MMFF94 calculation in Chem3D Pro 14.0 (Cambridge Soft: PerkinElmer Inc., Waltham, MA, USA). Molecular docking calculations were performed with AutoDock Vina and AutoDock Tools 1.5.6 (The Scripps Research Institute, La Jolla, California, United States of America) utilizing the hybrid Lamarckian Genetic Algorithm (LGA). The grid box size was 60 Å × 60 Å × 60 Å with 0.375 Å. A 3D protein–ligand complex structure was selected with the lowest energy (RMSD < 1.0) and visualized by PyMOL 2.5.7 (Schrodinger LLC, New York, NY, USA).

### 2.10 Colocalization Analysis

To analyse colocalization, we utilized the Colocalization Colormap plugin for ImageJ software. The plugin calculated the normalized mean deviation product (nMDP), which mathematically represents the correlation between intensities of corresponding pixels (values range from −1 to 1). We evaluated the index of correlation (IC), which represents the fraction of positively colocalized pixels in the analysed images [S5].

### 2.11 Statistical Analysis

All data are expressed as the mean ± the standard error of the mean (SEM) of independent experiments. Statistical analyses were conducted using GraphPad Prism 8.0 software. Statistical significance was determined using a one‐way ANOVA with Tukey's post hoc test for multigroup comparisons, while the non‐parametric Mann–Whitney *U* test was applied for two‐group comparisons where data did not consistently follow a normal distribution. *p < 0.05, **p < 0.01, ***p < 0.001 or ****p < 0.0001 were considered statistically significant.

## 3 Additional Information

Additional experimental procedures for cell culture, cell transfection, conditioned medium isolation, serum analysis, qPCR, Oil Red O staining, FA measurements, GEPIA2 database analysis,and protein–molecule binding affinity prediction, along with a list of chemical reagents, are provided in the Supporting Information.

## 4 Results

### 4.1 EA Restores Adipocyte Function in CM‐Stimulated White Adipocytes

**FIGURE 1:**
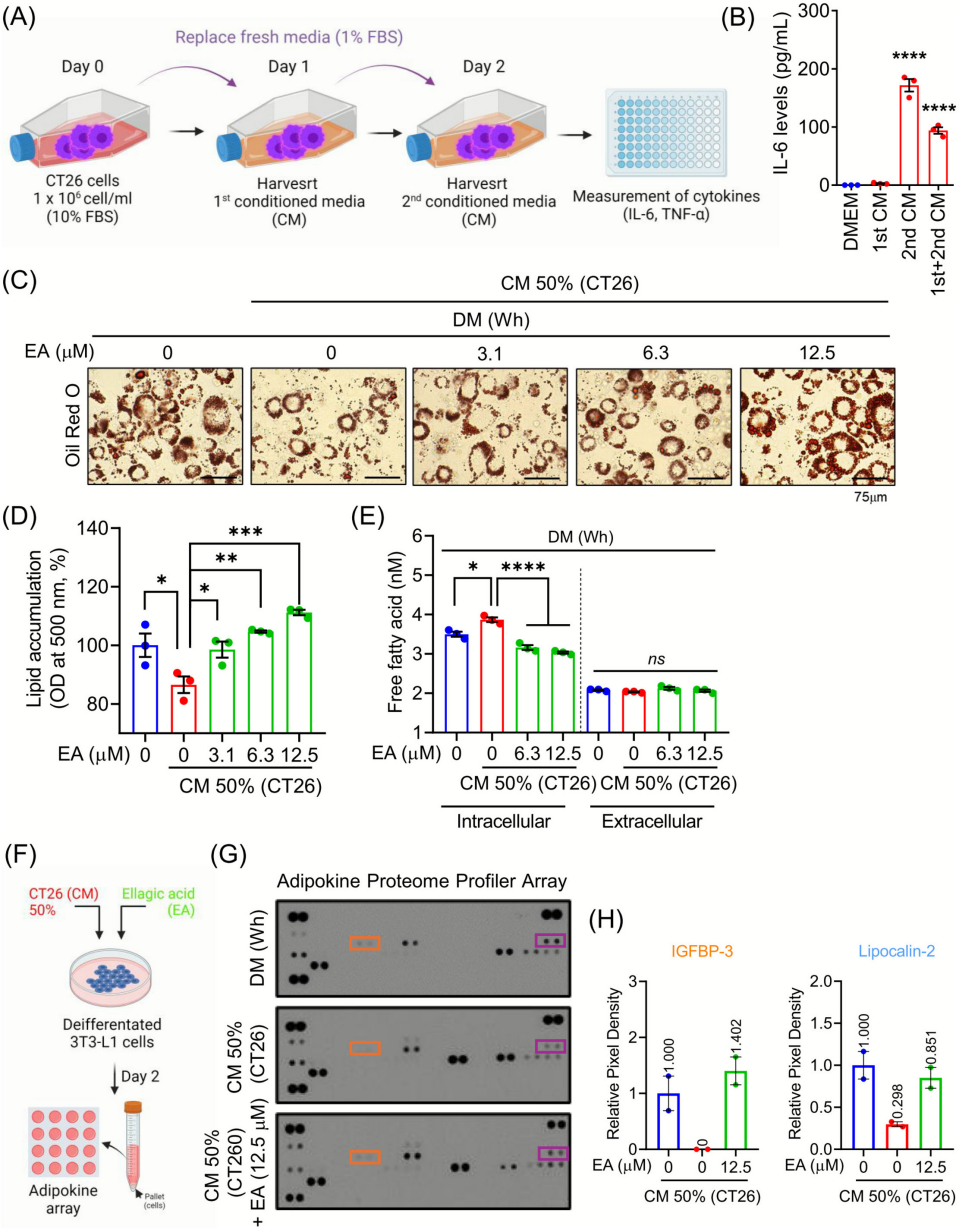
EA restores reduced adipocyte function in CM‐stimulated white adipocytes. (A) The experimental scheme for the preparation of the CT26 CM is shown. (B) IL‐6 levels were measured in CT26 CM with ELISA kits (*n* = 3). (C) Intracellular lipid droplets were stained with Oil Red O (magnification 400×, scale bar 75 = μm). (D) The quantification of intracellular lipid was detected at 500 nm in 3T3‐L1 cells differentiated into white adipocytes, and treated with 50% of CT26 CM or EA (3.1, 6.3 and 12.5 μM) (*n* = 3). (E) The levels of intracellular and extracellular free fatty acids were measured in 3T3‐L1 cells differentiated into white adipocytes, and treated with 50% of CT26 CM or EA (6.3 and 12.5 μM) (*n* = 3). (F, G) The expression of adipokines was analysed using a Mouse Adipokine Proteome Array kit. (H) The protein expression of IGFBP‐3 and lipocalin‐2 was analysed with ImageJ (*n* = 2). All data are expressed as the mean ± SEM. Statistical significance was determined using a one‐way ANOVA with Tukey's post hoc test for multigroup comparisons. *p < 0.05, **p < 0.01, ***p < 0.001 or ****p < 0.0001 were considered statistically significant. CM, conditioned medium. DM (Wh), differentiation medium. EA, ellagic acid.

To establish an in vitro model mimicking fat loss induced by cancer cachexia, we used CT26 mouse colon cancer cells. CM was collected from CT26 cells cultured under nutrient‐restricted conditions (1% FBS), and the pro‐inflammatory cytokines IL‐6 and TNF‐α were quantified (Figure 1A). CT26 cells cultured with 1% FBS for 2 days secreted IL‐6, but not TNF‐α (Figures 1B and S1A). Based on the results, we chose Day 2 (2nd CM) as the optimal time for collecting the CM. To determine the optimal CM concentration, Oil Red O staining was performed on white adipocytes treated with 25%, 50% or 75% CM. Lipid accumulation in white adipocytes was reduced at all CM concentrations, with the optimal condition achieved using a 1:1 mixture of CM and plain DMEM (CM 50%) (Figure S1B). To investigate whether EA protects against CM‐induced reduction in lipid accumulation, Oil Red O staining was performed and quantified in CM‐stimulated white adipocytes treated with EA (3.1, 6.3 and 12.5 μM). EA treatment significantly increased the lipid accumulation in CM‐stimulated white adipocytes (Figure 1C,D). To verify the restoration of the altered lipid metabolism in CM‐stimulated white adipocytes treated with EA, we extracted the crude lipid fraction from the cells, collected the supernatants and evaluated the amount of FA. The CM treatment in white adipocytes resulted in an increased intracellular FA, whereas the EA treatment restored the levels of FA to those of the white adipocytes. The extracellular levels of free FAs were not altered (Figure 1E). Given that adipokines regulate adipogenesis, adipocyte metabolism and function [S6], we performed adipoikine proteomic profiling using the Proteome Profiler Mouse Adipokine Array Kit to identify the alterations in adipokine production following CM treatment. The supernatant from CM–treated white adipocytes contained 13 different adipokines (Figure 1F,G and Table S1). Among these, three adipokines (endocan, RANTES and serpin E1) were increased, and two (IGFBP‐3 and lipocalin‐2) were decreased. We further investigated whether EA treatment can reverse these changes. EA restored the levels of IGFBP‐3 and lipocalin‐2, which were similar to those observed in white adipocytes (Figure 1H).

### 4.2 EA Rescues Reduced Adipogenesis by Upregulating PPARγ and RXRβ

**FIGURE 2:**
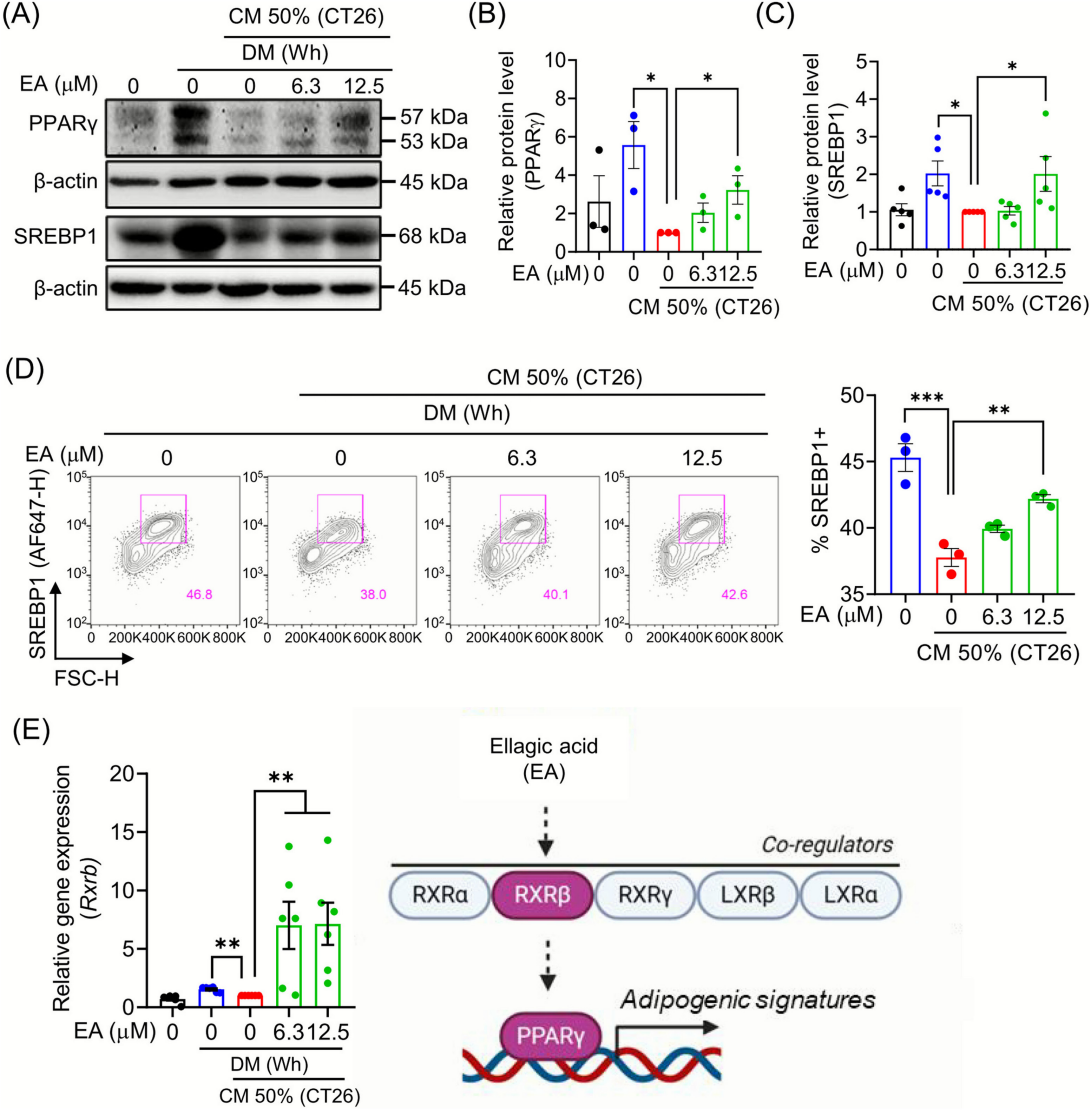
EA increases adipogenesis by upregulating RXRb and PPARγ. (A) Protein levels of PPARγ and SREBP1 were analysed using Western blot analysis. (B, C) Signal intensities of the protein bands were measured with ImageJ and normalized to the β‐actin (*n* = 3–5). (D) SREBP1‐positive cells were analysed with flow cytometry (*n* = 3). (E) Relative mRNA expression of *Rxrb* was determined by RT‐PCR, and data were normalized to *Gapdh* (*n* = 6). All data are expressed as the mean ± SEM. Statistical significance was determined using a one‐way ANOVA with Tukey's post hoc test for multigroup comparisons. *p < 0.05, **p < 0.01 or ***p < 0.001 were considered statistically significant. CM, conditioned medium. DM (Wh), differentiation medium. EA, ellagic acid.

To explore the potential mechanism of EA in restoring reduced adipogenesis in CM‐treated white adipocytes, we first investigated whether EA treatment regulates the protein levels of PPARγ and C/EBPα, which are fundamental transcription factors for adipogenesis. EA treatment increased the protein levels of PPARγ (Figure 2A,B) but did not change the level of C/EBPα (Figure S1C). It is well established that PPARγ induces the expression of SREBP1, a lipogenic transcription factor, which then triggers the production of the lipogenic enzymes. We next tested the potential impact of EA treatment on upregulating SREBP1 expression in CM‐treated white adipocytes. The EA treatment significantly increased the protein levels of SREBP1 (Figure 2A,C,D). To better understand the mechanisms by which EA promotes adipogenesis, we investigated whether EA treatment recruits coactivator genes, including *Rxra*, *Rxrb*, Rxrg, *Nr1h2* and *Nr1h3*, to regulate PPARγ transcriptional activity. CM treatment was found to reduce the mRNA expression of *Rxrb*, *Nr1h2* and *Nr1h3* compared to untreated white adipocytes. Importantly, the EA treatment increased the *Rxrb* mRNA expression (Figures 2E,F and S1D).

### 4.3 Upregulation of RXRβ and PPARγ by EA Increases Adipogenesis in CM‐exposed 3T3‐L1 cells

**FIGURE 3:**
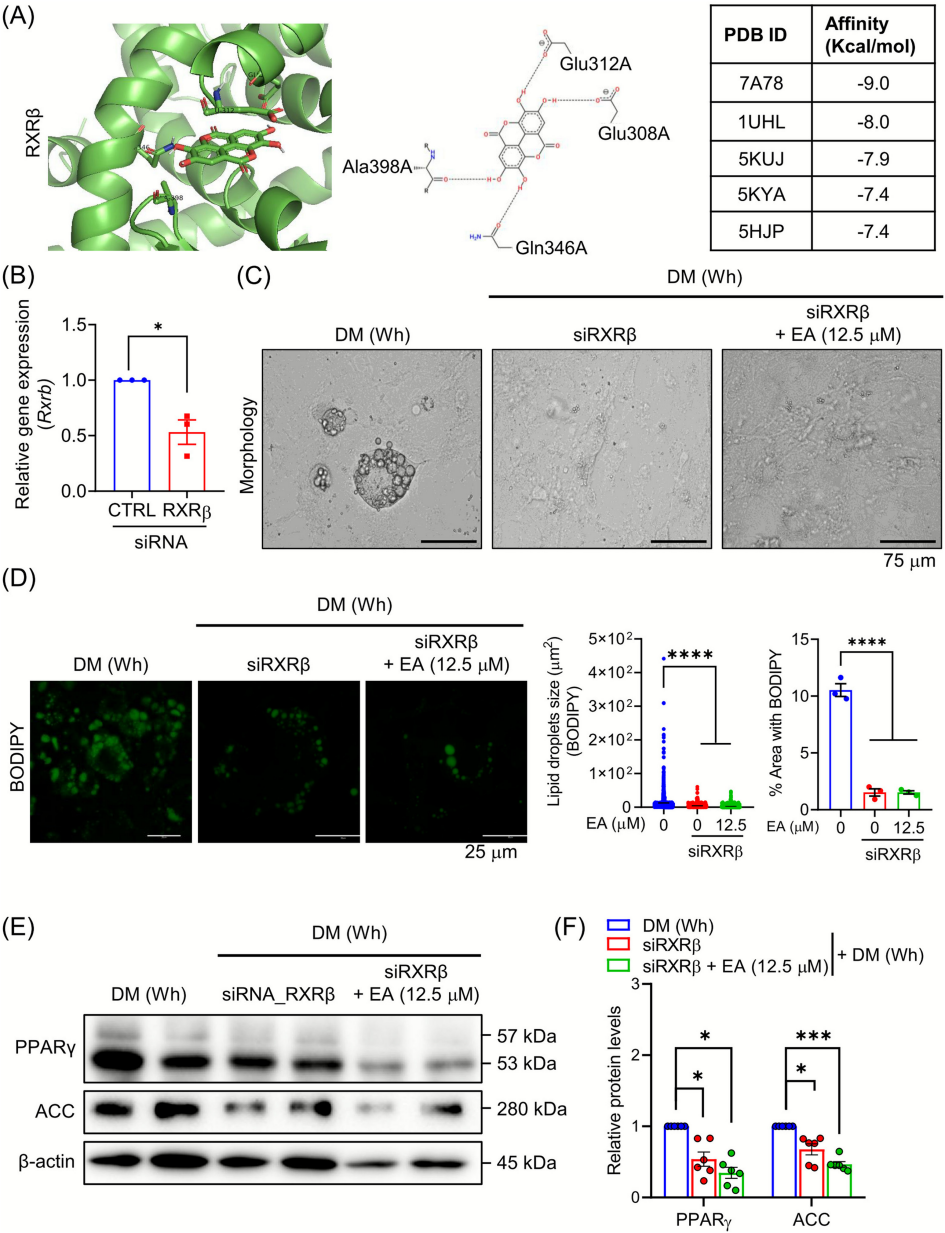
EA‐mediated RXRβ‐PPARγ axis activation mitigates the reduction of adipogenesis by CT26 CM. (A) Overview of the crystal structure of the complex between RXRB (PDB ID: 7A78) and EA, and expected intermolecular interactions are shown. (B) Relative mRNA expression of *Rxrb* was measured by RT‐PCR in 3T3‐L1 differentiated into white adipocytes with or without si*Rxrb*, and data are normalized to *Gapdh* (*n* = 3). (C) Representative morphological images of 3T3‐L1 treated with *siRxrb* and/or EA are shown (magnification 400×, scale bar = 75 μm). (D) The lipid droplets were stained with BODIPY‐Green (magnification 1000×, scale bar = 25 μm). Lipid droplet sizes and area were measured using the ImageJ software (*n* = 3). (E) Protein expression of PPARγ and ACC was measured by Western blot analysis. (F) Intensities of the protein bands were measured with ImageJ and normalized to β‐actin (*n* = 6). All data are expressed as the mean ± SEM. Statistical significance was determined using a one‐way ANOVA with Tukey's post hoc test for multigroup comparisons and the non‐parametric Mann–Whitney U test for two‐group comparisons. *p < 0.05, **p < 0.01 or ***p < 0.001 were considered statistically significant. CM, conditioned medium. DM (Wh), differentiation medium. EA, ellagic acid.

Because EA treatment increased the RXRβ gene expression, we evaluated the potential binding interaction between RXRβ and EA through molecular docking. The molecular docking simulations predicted a stable and high‐affinity interaction between EA and the active site of RXRβ, with predicted binding energies ranging from −7.4 to −9.0 kcal/mol across various protein crystal structures (PDB IDs: 7A78, 1UHL, 5KUJ, 5KYA and 5HJP). The predicted binding pose showed the compound fitting favourably into the binding pocket, stabilized by a network of key hydrogen bonds with residues Glu308, Glu312, Gln346 and Ala398A (Figure 3A). To investigate whether RXRβ regulation restores adipogenesis in CM‐treated white adipocytes, we inhibited RXRβ genetically using siRNA transfection (Figure 3B). Genetic inhibition of RXRβ blocked adipocyte differentiation, similar to the CM treatment, and was not rescued by EA treatment (Figure 3C). Furthermore, EA treatment failed to restore the intracellular lipid droplet content and size in knockdown of RXRβ in 3T3 cells, quantified by BODIPY staining (Figure 3D). EA treatment did not induce PPARγ and ACC protein expression in RXRβ siRNA‐treated cells, indicating that RXRβ is involved in regulating PPARγ and ACC transcription (Figure 2E,F). Database analysis revealed that mRNA expression of *RXRB* in human normal subcutaneous WAT was positively correlated with the levels of adipogenic gene signatures (*PPARG*, *CEBPA* and *PPARGC1A*), but not with lipogenic gene signatures (*ACLY*, *ACACA*, *FASN*, *SCD* and *SREBF1*) (Figure S1E).

### 4.4 EA Protects Against Weight Loss by Restoring Decreased White Fat Mass

**FIGURE 4:**
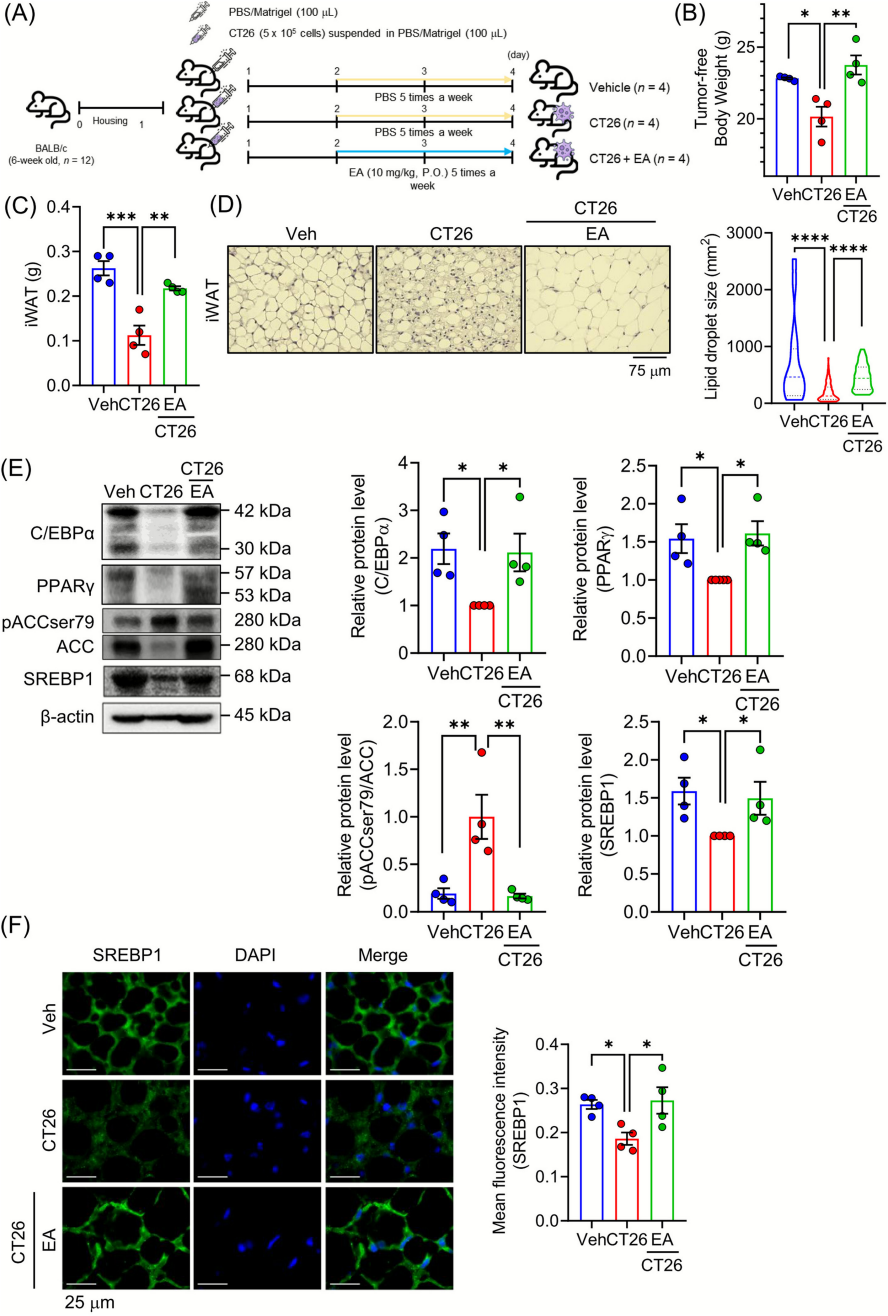
EA protects against body weight loss in CT26 tumour‐induced cachectic mice. (A) The experimental scheme of the in vivo study is shown. BALB/c mice were subcutaneously inoculated with 5 × 10^5^ CT26 cells (CT26 group), except for the vehicle group. EA administration (10 mg/kg) via oral gavage was started 1 week after tumour cell injection (CT26 + EA group). 0.9% Normal saline (vehicle group and CT26 group) or EA (CT26 + EA group) was fed five times per week for 2 weeks. (B) The tumour‐free weight was calculated by subtracting the isolated tumour weight from the body weight (*n* = 4). (C) The combined bilateral weight of the iWAT is shown (*n* = 4). (D) The H&E‐stained image of the iWAT (magnification 400×, scale bar = 75 μm) is shown, and lipid droplet sizes were calculated using the ImageJ software. (E) The protein levels of C/EBPα, PPARγ, and pACC and ACC were analysed by Western blot analysis. Signal intensities of the protein bands were measured with ImageJ and normalized to β‐actin (*n* = 4). (F) The paraffin‐embedded iWAT was stained with SREBP1 (green) and DAPI (blue) (magnification 1000×, scale bar = 25 μm), and representative images are shown. Fluorescence intensity of SREBP1 was quantified using the ImageJ software (*n* = 4). Data are expressed as the mean ± SEM. Statistical significance was determined using a one‐way ANOVA with Tukey's post hoc test for multigroup comparisons. *p < 0.05, **p < 0.01 or ***p < 0.001 were considered statistically significant. EA, ellagic acid. eWAT, epididymal white adipose tissue. iWAT, inguinal white adipose tissue.

To evaluate the effectiveness of the EA (10 mg/kg) treatment in reducing adipose tissue and body weight in a mouse model of colon cancer cachexia, we used the CT26 mouse colon cell line. CT26 cells were injected subcutaneously into the right flank of BALB/c mice, and EA was administered orally (Figure 4A). The EA treatment resulted in an increase in tumour‐free body weight without decreasing the weight of the tumour (Figures 4B and S2A,B). In addition, the weight of the GAS, TA and heart was not altered by the EA treatment (Figure S2C). While tumour induction led to an increased spleen weight and elevated serum IL‐6 levels, the EA treatment did not decrease either of these changes (Figure S2D,E). It did result in an increase in both iWAT and eWAT, suggesting that the restoration of body weight is associated with the increased mass of the iWAT (Figures 4C and S3A,B). Furthermore, the EA treatment increased the lipid droplet size of the iWAT (Figure 4D) and eWAT (Figure S3C,D). To elucidate the pharmacological mechanism of EA, we investigated the expression of lipogenic and adipogenic proteins in the iWAT. EA increased the adipogenic‐related protein levels of C/EBPα and PPARγ and the lipogenic‐related protein levels of SREBP1 in the iWAT (Figure 4E). EA treatment decreased ACC phosphorylation in the WAT (Figure 4E). However, EA treatment did not change the protein levels of PGC1α and FAS in the iWAT (Figure S3C,D). Subsequently, IF staining showed that the EA treatment increased the protein expression of SREBP1 in the iWAT but not the eWAT (Figures 4F and S3F). To further explore the effect of EA on lipolysis and thermogenesis, we examined the protein expression of ATGL (lipolysis) and UCP1 (thermogenesis) in the iWAT. The EA treatment did not change the protein expression of ATGL and UCP1 in the iWAT (Figure S3G).

Furthermore, we examined whether EA treatment systemically regulates lipogenesis through hepatic lipogenesis because the liver weight was increased in cachectic mice but remained unchanged following the EA treatment (Figure S4A). To evaluate hepatic histological changes caused by cachexia, we performed Masson's trichrome staining. It was shown that liver tissues in a cachectic state exhibit collagen and lipid droplet accumulation surrounding the hepatic central vein (Figure S4B). EA treatment reduced lipid droplet formation but did not decrease the fibrosis‐related mRNA expression of Col1a1 and Vim in the liver tissue (Figure S4C). In BAT, lipid droplet size was reduced in the cachectic state, but the EA treatment increased it (Figure S4D). The protein expression of UCP1 and PGC1a was not altered in the cachectic state and by the EA treatment (Figure S4E). We next sought to determine whether the effects of EA (10 mg/kg) were consistent in the LLC1‐induced cachexia mouse model. The body weight and average weekly food intake did not differ significantly between the LLC1 tumour‐bearing mice (LLC1) and tumour‐free control mice (Veh) (Figure S5A,B). The EA treatment did not change the tumour weight or increase the tumour‐free body weight (Figure S5C,D). In addition, the EA treatment did not increase the weight of the iWAT, eWAT, GAS and TA (Figure S5E–H).

### 4.5 EA Alleviates Adipose Wasting in a Murine Model of Cancer Cachexia Through Activation of RXRβ

**FIGURE 5:**
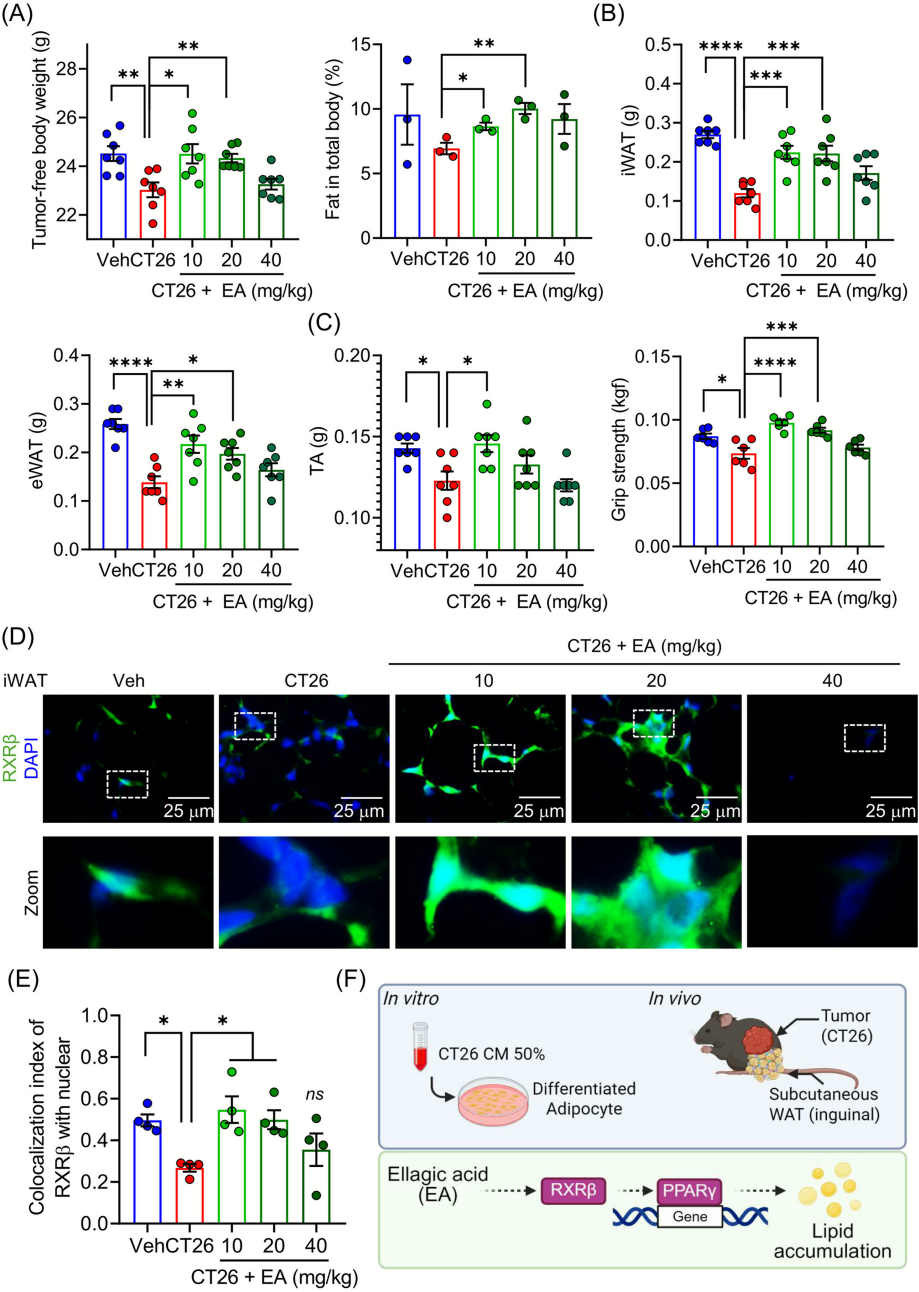
EA increases the expression of RXRβ in the iWAT of the CT26 cachexia model. (A) The tumour‐free weight was measured (*n* = 7), and the percentage of fat in the total body was measured with DEXA analysis (*n* = 3). (B) The combined bilateral weight of the iWAT and eWAT is shown (*n* = 7). (C) The combined bilateral weight of TA was measured, and grip strength was measured (*n* = 6–7). (D) The paraffin‐embedded iWAT was stained with RXRβ (green) and DAPI (blue) (magnification 1000×, scale bar = 25 μm), and representative images are shown. The bottom panels show zoomed views of the boxed areas in the top panels. (E) Index of correlation (IC) between RXRβ and nuclear (DAPI) was measured with the Colocalization Colormap plugin using ImageJ (*n* = 4). (F) Schematic of the experimental models and the mechanism of action for EA. Data are expressed as the mean ± SEM. Statistical significance was determined using a one‐way ANOVA with Tukey's post hoc test for multigroup comparisons. *p < 0.05, **p < 0.01 or ***p < 0.001 were considered statistically significant. EA, ellagic acid. eWAT, epididymal white adipose tissue. iWAT, inguinal white adipose tissue.

To investigate the dose‐dependent effects of EA on cancer‐induced cachexia, we treated CT26 tumour‐bearing mice with 10, 20 or 40 mg/kg of EA. The EA treatment did not significantly alter the total body weight, daily food intake or tumour weight (Figure S6A–C). Tumour‐free body weight was significantly increased in the 10 and 20 mg/kg groups. DEXA analysis confirmed a significant elevation in total body fat percentage (Figure 5A). The mass of both the iWAT and eWAT depots was markedly increased in the 10 and 20 mg/kg groups (Figure 5B). The EA treatment significantly increased the TA muscle mass but did not restore the GAS muscle mass. Furthermore, the EA treatment increased the grip strength compared to the CT26 group (Figures 5C and S6D). To validate the proposed mechanism of EA, we investigated whether EA treatment increased the protein expression of RXRβ in the iWAT. Indeed, EA treatment with 10 and 20 mg/kg increased the protein levels of RXRβ in the iWAT compared to the CT26 group (Figure 5D). Furthermore, the IC between RXRβ and the nucleus (marked by DAPI) was increased by the EA treatment, suggesting an increase in the nuclear colocalization of RXRβ in the iWAT (Figure 5E). Overall, EA ameliorates cancer cachexia‐induced fat loss by regulating the RXRβ–PPARγ pathway (Figure 5F).

## 5 Discussion

Adipose tissues are crucial for establishing energy homeostasis by regulating de novo lipogenesis (anabolism) and lipolysis (catabolism) [S7]. This homeostatic balance, however, could be impaired by aging and cancer [S8]. During cancer progression and development, glucose and lipid metabolism are often altered to cope with the elevated energy requirements of cancer cells [S9]. It is not surprising that glucose is primarily a fuel for rapidly proliferating tumours. Importantly, increasing evidence indicates that lipid metabolism is enhanced in metastatic cancer cells, which in turn activate actin remodelling and epigenetic processes that drive their transformation into mesenchymal‐like cancer cell types [18]. Indeed, inhibition of FA oxidation (FAO) in tumour cells could suppress metastasis and enhance the efficacy of chemotherapy (CT) and radiation (RT) [19]. In addition to increasing de novo lipogenesis, cancer cells also consume exogenous FAs from other tissues, such as adipose tissues and liver [20]. Secreted molecules, such as IL‐6, provoke metabolic disruption in adipose tissues by enhancing lipolysis, therefore releasing FAs into the bloodstream [21]. Thus, cancer cells undergoing nutritional stress and transitioning to metastasis could obtain their energy from both endogenous and exogenous FAs, resulting in increased fat loss in the body [22]. In the present study, we demonstrated that EA treatment not only reestablished lipid accumulation and suppressed lipid breakdown induced by CT26 CM but also protected against the loss of both iWAT and eWAT in CT26 tumour‐bearing mice.

Disruption of the adipogenesis process has been linked to the wasting of adipose tissue mass in cancer patients. In cancer cachexia, a significant reduction in the gene transcription of PPARγ, C/EBPα and SREBP‐1c has been reported in the eWAT of mice. Moreover, PPARγ mRNA expression in mesenteric adipose tissue (MEAT) and retroperitoneal adipose tissue (RPAT) significantly declined in tumour‐bearing rats beginning on Day 4 after tumour cells were injected [23]. However, the reprogramming of subcutaneous WAT in cancer cachexia remains unclear. To further our understanding, we evaluated the expression of adipogenic genes in the iWAT (subcutaneous WAT) of CT26 tumour‐bearing mice. Protein expression of PPARγ, C/EBPα and PGC1α was reduced in the iWAT; however, EA treatment restored their expression. Interestingly, ACC phosphorylation was elevated in the iWAT. The phosphorylation of ACC limits the activity of de novo FA synthesis and terminates lipid synthesis. AMP‐activated protein kinase (AMPK) could phosphorylate and inhibit ACC, enhancing FAO and facilitating ATP generation [24]. The activation of the AMPK‐dependent pathway is recognized for its association with enhanced lipolysis in adipose tissue loss of cachexia experiment models [25]. Pharmacological AMPK activation by AICAR and metformin inhibits PPARs by inhibiting the PPRE reporter activity. In contrast, AMPK inhibitor compound C increases the basal and rosiglitazone‐stimulated PPARγ activity in hepatoma cells [26]. In line with this, regulating the balance between AMPK and PPARγ is crucial for lipid homeostasis and might protect against fat loss in cancer cachexia.

Nuclear receptors, which include RXRβ, are recognized for their role in lipid metabolism, predominantly through their interaction with PPARγ. RXRs and PPARγ form heterodimers that modulate the expression of genes crucial for adipocyte development and fat storage [27]. Upon binding of RXRβ to PPARγ, synergistic activation of genes occurs, facilitating the differentiation of preadipocytes into mature adipocytes and enhancing the fat storage capacity of adipose tissues [28]. EA‐induced upregulation of RXRβ enhances PPARγ expression and promotes lipid accumulation in CT26 CM–treated white adipocytes. It is supported by the finding that EA treatment restores the iWAT and eWAT mass in CT26 tumour‐bearing mice. These findings suggest that upregulation of RXRβ is a possible therapeutic mechanism for combating fat loss in cancer cachexia.

In addition, adipokines are cell‐signalling proteins released by adipose tissue that contribute to maintaining adipogenesis and adipocyte metabolism [29]. Based on adipokine arrays, CT26 CM exposure altered adipokine secretion patterns in white adipocytes. The levels of IGFBP‐3 and lipocalin‐2 were reduced in CT26 CM–treated white adipocytes, whereas EA treatment restored their secretion to levels comparable to those of control white adipocytes. IGFBP‐3 is known to be involved in the differentiation of adipocytes and the maintenance of mature adipocytes, which regulates glucose uptake by modulating insulin signalling [30]. Likewise, lipocalin‐2 is associated with adipogenesis in a C/EBPs–dependent manner [31]. Although many studies have shown that excessive IGFBP‐3 and lipocalin‐2 may induce insulin resistance, diabetes and tumorigenesis, genetic deletion of lipocalin‐2 reduces adipogenic gene expression, and IGFBP‐3 can function as a tumour suppressor [32, 33]. Overall, further work is required to firmly understand the complexities of both IGFBP‐3 and lipocalin‐2 in adipose tissues around tumours.

Several studies have reported the pharmacokinetics, safety and metabolism of EA in humans. Free EA, not EA‐containing foods, can be detected in the plasma 2 h after ingestion [34]. EA is metabolized by the human intestinal microbiota into urolithin derivatives, with urolithin A and B conjugates being the most detectable forms in plasma, urine and various tissues in both animals and humans [35]. They reach maximum concentrations in plasma 24–48 h after ingestion of EA [34]. Although dose translation of EA from animals to humans has not been fully investigated, some clinical studies have administered 180–200 mg of EA and demonstrated its clinical relevance in hormone‐refractory prostate cancer (HRPC) [36], irritable bowel syndrome [37] and non‐alcoholic fatty liver disease [38]. Although the effect of EA on cancer cachexia‐induced fat and muscle atrophy has not been investigated, a few studies have indicated that urolithin A supplementation at doses of either 500 or 1000 mg enhanced mitochondrial gene expression in the skeletal muscles and improved cellular health after a 4‐week oral administration in sedentary older adults [39]. Singh et al. investigated the impact of oral supplementation with urolithin A in a randomized clinical trial in middle‐aged adults. The results showed improvements in muscle strength and exercise performance [S10]. Considering that a cohort study linked low skeletal muscle status to increased postoperative complications and mortality [40], our finding that EA supplementation mitigated muscle loss suggests that this strategy may promote more favourable outcomes (e.g., time to recovery, complications and short/long‐term mortality) by preserving muscle mass in humans undergoing colon cancer surgery. Taken together, EA administration leads to the production of its active metabolite, urolithin A, suggesting potential benefits for improving muscle function in cancer cachexia models.

This study has several limitations. Seven‐week‐old mice are still in a growth and maturation phase and may not fully reflect the condition of human cancer in older age. The use of fresh DMEM as a control medium does not control for the depletion of nutrients (e.g., glucose) and the accumulation of metabolites that occur in the conditioned medium. An adipocyte‐specific RXRβ knockout mouse model is necessary to establish the protective role of RXRβ.

In conclusion, this study demonstrates that EA supplementation can mitigate fat mass and body weight loss during the progression of cancer cachexia. We found that EA activates the RXRβ–PPARγ pathway, which enhances de novo lipogenesis in both CT26 CM–treated white adipocytes and in the iWAT of CT26 tumour‐bearing mice. Taken together, our findings highlight that activating the RXRβ–PPARγ pathway is a potential mechanism for preserving fat mass and body weight against cancer cachexia‐induced malnutrition.

## Funding

This study was funded by a National Research Foundation of Korea (NRF) grant funded by the Korean government (MSIP) (NRF‐2021R1A2C2010460, NRF‐2022R1A2C2005930 and RS‐2025‐02243112) and Basic Science Research Program through the National Research Foundation of Korea (NRF) funded by the Ministry of Education (RS‐2024‐00461726). This work was supported by a grant from Kyung Hee University in 2025 (KHU‐20251294).

## Conflicts of Interest

The authors declare no conflicts of interest.

## Supporting information


**Figure S1:** (A) The level of TNF‐α was measured (*n* = 3). (B) Lipid accumulation was measured using Oil Red O staining according to the percentage of conditioned medium (*n* = 3). (C) Protein levels of C/EBPα were analysed using Western blot analysis. β‐actin was used as a loading control (*n* = 3). (D) Relative mRNA expression of *Nr1h3*, *Nr1h2*, *Rxra* and *Rxrg* was measured by RT‐PCR (*n* = 5). (E) Scatter plots show the correlation between RXRB and adipogenic gene signature (PPARG, CEBPA and PPARGC1A) genes or between RXRB and lipogenic gene signature (ACLY, ACACA, FASN, SCD and SREBF1) according to Pearson correlation analysis in human normal subcutaneous WAT (GEPIA2 database). All data are expressed as the mean ± SEM of data from three or more separate experiments. Statistical significance was determined using a one‐way ANOVA with Tukey's post hoc test for multigroup comparisons. *p < 0.05 was considered statistically significant. CM, conditioned medium. DM (Wh), differentiation medium. EA, ellagic acid.
**Figure S2:** (A) The body weight was measured every week after CT26 tumour cells were injected into mice (*n* = 4). (B) Representative images of tumour and tumour weight were indicated (*n* = 4). The weight of (C) skeletal and cardiac muscles, and (E) spleen was shown (*n* = 4). (F) The level of IL‐6 in serum was detected with anti‐IL‐6 ELISA kit (*n* = 4). All data are expressed as the mean ± SEM. Statistical significance was determined using a one‐way ANOVA with Tukey's post hoc test for multigroup comparisons and the non‐parametric Mann–Whitney *U* test for two‐group comparisons. *p < 0.05 or **p < 0.01 were considered statistically significant. EA, ellagic acid. IL‐6, interleukin‐6. Veh, vehicle.
**Figure S3: (**A) Representative images of iWAT and eWAT were shown. (B) Weight of eWAT was measured (*n* = 4). (C) The H&E staining image was represented in eWAT (magnification 400×, scale bar = 75 μm) and (D) lipid droplet sizes were calculated using ImageJ software. (E) The protein levels of PGC1α and FAS were analysed using Western blot analysis (*n* = 5). β‐actin was used as a loading control. (F) Representative immunofluorescence images of SREBP1 in eWAT were shown (magnification 400×, scale bar 75 = μm). Fluorescence intensity was analysed with Image J software (*n* = 4). (G) Protein levels of ATGL and UCP1 in iWAT were shown (*n* = 4). All data are expressed as the mean ± SEM. Statistical significance was determined using a one‐way ANOVA with Tukey's post hoc test for multigroup comparisons. *p < 0.05, **p < 0.01 or ^****^ *p* < 0.0001 were considered statistically significant. EA, ellagic acid. eWAT, epididymal white adipose tissue. iWAT, inguinal white adipose tissue. Veh, Vehicle.
**Figure S4:** (A) The weight of liver was shown (*n* = 4). (B) Representative Masson's Trichrome images of liver were shown (magnification 200×, scale bar 150 = μm). (C) Relative mRNA expression of *Col1a1* and *Vim* was measured by RT‐PCR (*n* = 4). (D) Representative H&E images of iBAT were shown (magnification 400×, scale bar 75 = μm). (E) Lipid droplet sizes were measured with Image J software. (E) Protein levels of UCP1 and PGC1α were analysed by Western blot analysis (*n* = 4). All data are expressed as the mean ± SEM. Statistical significance was determined using a one‐way ANOVA with Tukey's post hoc test for multigroup comparisons. *p < 0.05 or **p < 0.01 were considered statistically significant. EA, ellagic acid. iBAT, interscapular brown adipose tissue. Veh, Vehicle.
**Figure S5:** (A) Body weight was measured (*n* = 7). (B) Weekly food intake was measured (*n* = 7). (C) Weight of tumour was measured. (D) Tumour free body weight was measured (*n* = 7). (E–H) Weight of iWAT, eWAT, GAS and TA was measured (*n* = 7). All data are expressed as the mean ± SEM. Statistical significance was determined using a one‐way ANOVA with Tukey's post hoc test for multigroup comparisons and the non‐parametric Mann–Whitney *U* test for two‐group comparisons. *p < 0.05, ***p < 0.05 or ^****^ *p* < 0.0001 were considered statistically significant. EA, ellagic acid. eWAT, epididymal white adipose tissue. GAS, gastrocnemius. iBAT, interscapular brown adipose tissue. iWAT, inguinal white adipose tissue. TA, tibialis anterior. Veh, vehicle.
**Figure S6:** (A) Body weight was measured (*n* = 7). (B) Daily food intake was measured. (C, D) Weight of tumour, and GAS was measured (*n* = 7). All data are expressed as the mean ± SEM. Statistical significance was determined using a one‐way ANOVA with Tukey's post hoc test for multigroup comparisons. **p < 0.01 was considered statistically significant. EA, ellagic acid. GAS, gastrocnemius. Veh, vehicle.
**Table S1:** The expression of adipokines. Protein expression of adipokines was measured by Western blot analysis. Symbol (#) indicated experimental number (*n* = 2). Symbol (*) indicated protein expression and all data are analysed with Image J software. CM, conditioned medium, DM (Wh), differentiation medium (white adipocyte), EA, ellagic acid.
**Table S2:** Information on antibodies for Western blot and immunofluorescence staining.
**Table S3:** Information on primers for RT‐PCR. *Acaca:* acetyl‐CoA carboxylase alpha, *Cebpa:* CCAAT enhancer binding protein alpha, *Fasn:* fatty acid synthase, *Gapdh:* glyceraldehyde‐3‐phosphate dehydrogenase, *Nr1h2:* nuclear receptor subfamily 1 group h member 2, *Nrih3:* nuclear receptor subfamily 1 group h member 3, *Pparg:* peroxisome proliferator activated receptor gamma, *Rxra:* retinoid X receptor alpha, *Rxrb:* retinoid X receptor beta, *Rxrg:* retinoid X receptor gamma, *srebf1:* sterol regulatory element binding transcription factor 1, *Srebf2:* sterol regulatory element binding transcription factor 2.

## Data Availability

All data are available on request.
