# Supplementary material for: Ellagic Acid Alleviates Abnormal Fat Reduction by Activating the RXRβ–PPARγ Pathways in a CT26 Tumour‐Induced Cachexia Mouse Model
Source: J Cachexia Sarcopenia Muscle. 2026 Jan 28;17(1):e70176. doi: 10.1002/jcsm.70176 (PMC12848599; doi:10.1002/jcsm.70176)
Supplement: Supplementary file 1 — Figure S1: (A) The level of TNF‐α was measured (n = 3). (B) Lipid accumulation was measured using Oil Red O staining according to the percentage of conditioned medium (n = 3). (C) Protein levels of C/EBPα were analysed using Western blot analysis. β‐actin was used as a loading control (n = 3). (D) Relative mRNA expression of Nr1h3, Nr1h2, Rxra and Rxrg was measured by RT‐PCR (n = 5). (E) Scatter plots show the correlation between RXRB and adipogenic gene signature (PPARG, CEBPA and PPARGC1A) genes or between RXRB and lipogenic gene signature (ACLY, ACACA, FASN, SCD and SREBF1) according to Pearson correlation analysis in human normal subcutaneous WAT (GEPIA2 database). All data are expressed as the mean ± SEM of data from three or more separate experiments. Statistical significance was determined using a one‐way ANOVA with Tukey's post hoc test for multigroup comparisons. *p < 0.05 was considered statistically significant. CM, conditioned medium. DM (Wh), differentiation medium. EA, ellagic acid. Figure S2: (A) The body weight was measured every week after CT26 tumour cells were injected into mice (n = 4). (B) Representative images of tumour and tumour weight were indicated (n = 4). The weight of (C) skeletal and cardiac muscles, and (E) spleen was shown (n = 4). (F) The level of IL‐6 in serum was detected with anti‐IL‐6 ELISA kit (n = 4). All data are expressed as the mean ± SEM. Statistical significance was determined using a one‐way ANOVA with Tukey's post hoc test for multigroup comparisons and the non‐parametric Mann–Whitney U test for two‐group comparisons. *p < 0.05 or **p < 0.01 were considered statistically significant. EA, ellagic acid. IL‐6, interleukin‐6. Veh, vehicle. Figure S3: (A) Representative images of iWAT and eWAT were shown. (B) Weight of eWAT was measured (n = 4). (C) The H&E staining image was represented in eWAT (magnification 400×, scale bar = 75 μm) and (D) lipid droplet sizes were calculated using ImageJ software. (E) The protein levels of [file JCSM-17-e70176-s001.docx]

**Supplementary figure 1.** (A) The level of TNF-α was measured (*n* = 3). (B) Lipid accumulation was measured using Oil Red O staining according to the percentage of conditioned medium (*n* = 3). (C) Protein levels of C/EBPα were analyzed using Western blot analysis. β-actin was used as a loading control (*n* = 3). (D) Relative mRNA expression of *Nr1h3*, *Nr1h2*, *Rxra*, and *Rxrg* was measured by RT-PCR (*n* = 5). (E) Scatter plots show the correlation between RXRB and adipogenic gene signature (PPARG, CEBPA, and PPARGC1A) genes or between RXRB and lipogenic gene signature (ACLY, ACACA, FASN, SCD, and SREBF1) according to Pearson correlation analysis in human normal subcutaneous WAT (GEPIA2 database). All data are expressed as the mean ± S.E.M. of data from three or more separate experiments. Statistical significance was determined using a one-way ANOVA with Tukey's *post-hoc* test for multi-group comparisons. ^*^*p* < 0.05 was considered statistically significant. DM (Wh), differentiation medium. CM, conditioned medium. EA, ellagic acid.

**Supplementary figure 2.** (A) The body weight was measured every week after CT26 tumor cells were injected into mice (*n* = 4). (B) Representative images of tumor and tumor weight were indicated (*n* = 4). The weight of (C) skeletal and cardiac muscles, and (E) spleen was shown (*n* = 4). (F) The level of IL-6 in serum was detected with anti-IL-6 ELISA kit (n = 4). All data are expressed as the mean ± S.E.M. Statistical significance was determined using a one-way ANOVA with Tukey's *post-hoc* test for multi-group comparisons and the non-parametric Mann-Whitney *U* test for two-group comparisons. ^*^*p* < 0.05, or ^**^*p* < 0.01 were considered statistically significant. Veh, Vehicle. EA, ellagic acid. IL-6, Interleukin-6.

**Supplementary figure 3. (**A) Representative images of iWAT and eWAT were shown. (B) Weight of eWAT was measured (*n* = 4). (C) The H&E staining image was represented in eWAT (magnification 400×, scale bar = 75 μm) and (D) lipid droplet sizes were calculated using ImageJ software. (E) The protein levels of PGC1α and FAS were analyzed using Western blot analysis (*n* = 5). β-actin was used as a loading control. (F) Representative immunofluorescence images of SREBP1 in eWAT were shown (magnification 400×, scale bar 75 = μm). Fluorescence intensity was analyzed with Image J software (*n* = 4). (G) Protein levels of ATGL and UCP1 in iWAT were shown (*n* = 4). All data are expressed as the mean ± S.E.M. Statistical significance was determined using a one-way ANOVA with Tukey's *post-hoc* test for multi-group comparisons. ^*^*p* < 0.05, ^**^*p* < 0.01, or ^****^*p* < 0.0001 were considered statistically significant. Veh, Vehicle. EA, ellagic acid. eWAT, epididymal white adipose tissue. iWAT, inguinal white adipose tissue.

**Supplementary figure 4.** (A) The weight of liver was shown (*n* = 4). (B) Representative Masson’s Trichrome images of liver were shown (magnification 200×, scale bar 150 = μm). (C) Relative mRNA expression of *Col1a1* and *Vim* was measured by RT-PCR (*n* = 4). (D) Representative H&E images of iBAT were shown (magnification 400×, scale bar 75 = μm). (E) Lipid droplet sizes were measured with Image J software. (E) Protein levels of UCP1 and PGC1α were analyzed by Western blot analysis (*n* = 4). All data are expressed as the mean ± S.E.M. Statistical significance was determined using a one-way ANOVA with Tukey's *post-hoc* test for multi-group comparisons. ^*^*p* < 0.05, or ^**^*p* < 0.01 were considered statistically significant. Veh, Vehicle. EA, ellagic acid. iBAT, interscapular brown adipose tissue.

**Supplementary figure 5.** (A) Body weight was measured (*n* = 7). (B) Weekly food intake was measured (*n* = 7). (C) Weight of tumor was measured. (D) Tumor free body weight was measured (*n* = 7). (E-H) Weight of iWAT, eWAT, GAS, and TA was measured (*n* = 7). All data are expressed as the mean ± S.E.M. Statistical significance was determined using a one-way ANOVA with Tukey's *post-hoc* test for multi-group comparisons and the non-parametric Mann-Whitney *U* test for two-group comparisons. ^*^*p* < 0.05, ^***^*p* < 0.05 or ^****^*p* < 0.0001 were considered statistically significant. Veh, Vehicle. EA, ellagic acid. iBAT, interscapular brown adipose tissue. Veh, Vehicle. EA, ellagic acid. eWAT, epididymal white adipose tissue. iWAT, inguinal white adipose tissue. GAS, Gastrocnemius. TA, Tibialis anterior.

**Supplementary figure 6.** (A) Body weight was measured (*n* = 7). (B) Daily food intake was measured. (C-D) Weight of tumor, and GAS was measured (*n* = 7). All data are expressed as the mean ± S.E.M. Statistical significance was determined using a one-way ANOVA with Tukey's *post-hoc* test for multi-group comparisons. ^**^*p* < 0.01 was considered statistically significant. Veh, Vehicle. EA, ellagic acid. GAS, Gastrocnemius.

**Supplementary Table 1. The expression of adipokines.** Protein expression of adipokines was measured by Western blot analysis. Symbol (#) indicated experimental number (*n* = 2). Symbol (*) indicated protein expression and all data are analyzed with Image J software. DM (Wh), differentiation medium (white adipocyte), CM, conditioned medium, EA, ellagic acid.

**Supplementary Table 2.** Information on antibodies for Western blot and immunofluorescence staining.

**Supplementary Table 3.** Information on primers for RT-PCR. *Acaca:* acetyl-CoA carboxylase alpha, *Cebpa:* CCAAT enhancer binding protein alpha, *Fasn:* fatty acid synthase, *Gapdh:* glyceraldehyde-3-phosphate dehydrogenase, *Nr1h2:* nuclear receptor subfamily 1 group h member 2, *Nrih3:* nuclear receptor subfamily 1 group h member 3, *Pparg:* peroxisome proliferator activated receptor gamma, *Rxra:* retinoid X receptor alpha, *Rxrb:* retinoid X receptor beta, *Rxrg:* retinoid X receptor gamma, *srebf1:* sterol regulatory element binding transcription factor 1, *Srebf2:* sterol regulatory element binding transcription factor 2
